# Lessons Learned From a Collaborative to Improve Care for Patients With Diabetes in 17 Community Health Centers, Massachusetts, 2006

**Published:** 2010-06-15

**Authors:** Celeste A. Lemay, Brianne M. Beagan, Warren J. Ferguson, J. Lee

**Affiliations:** University of Massachusetts Medical School; Massachusetts Department of Public Health, Boston, Massachusetts; University of Massachusetts Medical School, Worcester, Massachusetts; University of Massachusetts Medical School, Worcester, Massachusetts

## Abstract

**Introduction:**

In 2006, the Massachusetts League of Community Health Centers convened a collaborative to systematically improve health care delivery for patients with diabetes in 17 community health centers. Our goal was to identify facilitators of and barriers to success reported by teams that participated in this collaborative.

**Methods:**

The collaborative's activities lasted 13 months. At their conclusion, we interviewed participating team members. We asked about their teams' successes, challenges, and take-home messages for future collaborative efforts. We organized their responses into common themes by using the Chronic Care Model as a framework.

**Results:**

Themes that emerged as facilitators of success included shifting clinic focus to more actively involve patients and to promote their self-management; improving the understanding and implementation of professional guidelines; and expanding staff roles to accommodate these goals. Patient registries were perceived as beneficial but lacking adequate technical support. Other barriers were staffing and time constraints.

**Conclusions:**

Cooperative efforts to improve health care delivery for people with diabetes may benefit from educating the health care team about guidelines, establishing a stronger role for the patient as part of the health care team, and providing adequate technical instruction and support for the use of clinical databases.

## Introduction

Nearly 21 million people in the United States have diabetes, and prevalence is particularly high among racial/ethnic minorities and populations with low socioeconomic status (1). Community health centers (CHCs) serve more than 15 million patients annually, 64% of whom are from racial/ethnic minority populations and 92% of whom have incomes below 200% of federal poverty level (2). CHCs are thus ideal settings for monitoring and improving the quality of diabetes care and reducing diabetes-related health disparities. In April 2006, the Massachusetts League of Community Health Centers initiated the Massachusetts Diabetes Health Disparities Collaborative, a state-based quality improvement initiative for 17 CHCs (3). For 13 months, participating CHCs focused on achieving system change in the delivery of primary health care for patients with diabetes by applying the Chronic Care Model (4). The 6 components of the Chronic Care Model are self-management, decision support, delivery system design, clinical information, organization, and community. When fully functioning, these components constitute a system that encourages high-quality chronic disease management, productive interactions with patients who take an active part in their care, and support for providers with resources and expertise (5).

Although several studies (6-11) report on the success of methods used by collaboratives, as measured by outcomes data, few (6,12,13) have reported specific, detailed strategies for achieving success and overcoming challenges. Wang et al (12) informally interviewed collaborative team leaders at 2 of 4 CHCs participating in the North Carolina Diabetes Collaborative and found that senior leadership support, physician champions, multidisciplinary teams, and making the collaborative a priority for the health care team were factors for success. Barriers were staff turnover, lack of senior leadership and physician support, and low priority in the organization's strategic planning. Chin et al (6) conducted semistructured telephone interviews with representatives of CHCs in the Midwest that participated in the National Health Disparities Collaborative. The findings were similar to those of Wang et al (12). Major perceived successes included development of patient registries, improved diabetes care, improved adherence to standards, and increased patient and provider awareness. Building on these findings, in 2008 Chin et al (13) reported the results of a self-administered questionnaire completed by 1,006 participants at 165 health centers in 21 states. The barriers reported by respondents supported findings from the earlier study, including time, funding, and lack of staff.

We sought to identify strategies that contributed to the CHCs' successes and challenges encountered in the Massachusetts collaborative and to frame them in terms of the components of the Chronic Care Model. Our objective was to find specific, detailed strategies for improvement.

## Methods

Of the 52 CHCs in Massachusetts, 17 participated in the collaborative after receiving information and an invitation from the Massachusetts League of Community Health Centers. The 17 represented CHCs from across the state and both rural (18%) and urban (82%) settings. Each CHC assembled a team to participate in the collaborative. At a minimum, team members consisted of a provider champion (a primary care provider); a team leader, responsible for coordinating and reporting activities; and a data entry person, responsible for establishing, maintaining, and updating the electronic registries of diabetes patients. Teams implemented the Chronic Care Model using the provider champion's panel of diabetic patients, called the population of focus. We contacted team leaders at the end of the initiative in May 2007 and interviewed them using a site visit interview tool developed for this project. The tool is based on the ecological systems theory (14), which recognizes nested environmental systems and the interactions within and between the systems. Members of the collaborative's evaluation team reviewed questions for appropriateness, relevance, and comprehension and revised questions accordingly (Appendix). Study protocols were approved by the University of Massachusetts Medical School Committee for the Protection of Human Subjects in Research.

Two experienced interviewers collected data from all 17 sites. They interviewed team leaders and other team members who were available at the time of the interview during a site visit (9 sites) or by telephone. Interviews lasted 30 to 45 minutes. Interviewers took notes and transcribed them after each interview.

We used Microsoft Excel (Microsoft Corporation, Redmond, Washington) to summarize the data and facilitate content analysis (15,16). The 6 components of the Chronic Care Model formed the initial framework for the coding scheme. Responses were coded independently by 2 investigators, and intercoder agreement was 96%. Disputed responses were reviewed until 100% agreement was reached. We developed a secondary coding scheme to identify emergent subthemes for each Chronic Care Model component by using answers to the original interview questions and spontaneous comments. Subtheme responses were considered dominant if they were reported by multiple CHC teams. Two investigators working independently conducted a second round of coding using the subtheme coding tool. Intercoder agreement was 88%. A third investigator reviewed disputed responses until 100% agreement was reached.

Participating CHCs uploaded monthly reports and data summaries to a password-protected Internet database. These reports included information about plan-do-study-act (PDSA) cycles, a quality improvement model. PDSA cycles are useful for rapidly testing a proposed change in procedure or practice by developing a plan to test the change (plan), testing the change (do), evaluating the tested change (study), and determining what adjustments are needed (act) (17). We audited these monthly reports and collected information pertaining to the number of PDSA cycles and the Chronic Care Model component they addressed.

## Results

Selected characteristics of patients in participating CHCs compared with those in the general state population (Table 1) suggest that CHCs serve a more ethnically diverse population (18) with a higher prevalence of uninsurance (19). Interviewees were team leaders from all 17 CHCs, 2 provider champions, and 7 team members. Each component of the Chronic Care Model was represented in the findings (Table 2).

### Self-management

Teams reported that their patients were more likely to set self-management goals as a result of the CHC's participation in the collaborative. Many reported that changing the practice philosophy at the CHC — specifically, recognizing that patients play a role in determining their health and can be empowered to control their disease — was necessary to achieve this success. Allowing patients to assume some responsibility for the management of their diabetes was a new approach for many teams:

The empowerment of people to do self-management goal setting, this was something that we were not aware of before [the collaborative began]. — CHC 16, team leader

Teams noted that helping patients set goals for self-management became a priority for the entire health care team at the CHC. They approached patients as a team, followed up with patients who had not yet set a goal, and met with patients before and after their clinic visit:

We introduced the topic to our patients. We called patients to discuss it. We discussed it at group visit meetings. We used [team members] to implement self-management goal setting with our patients. [Dr A] was very proactive in discussing self-management with her patients; [Dr B] wanted people to go home and think about it. The [medical assistant] would follow up with these patients via telephone and help them set goals. — CHC 16, team leader

### Decision support

Understanding and implementing clinical guidelines for diabetes emerged as a major theme under the decision support component of the Chronic Care Model. Teams agreed that structured learning sessions (three 2-day conferences organized by the League of Community Health Centers during the collaborative that provided CHCs opportunities to share best practices) were instrumental in updating care to be consistent with scientific evidence and patient preferences. Understanding guidelines provided the impetus to improving patient care:

Being involved in the collaborative has drastically improved the quality of care that our diabetic patients receive. When a diabetic patient comes in, we know what the standards are. We offer our patients more, such as group meetings, healthy choices, relationships. We have become involved in other initiatives, increased our knowledge, and are committed to the project. — CHC 16, team leader

### Delivery system design

Teams noted that patients' language and literacy issues were a challenge to changing delivery system design. Strategies for addressing this challenge were recruiting multilingual staff, adapting and translating materials, redesigning educational handouts using a pictorial focus, and using interpreters.

Teams reported the need to expand the role of health care providers, particularly medical assistants and nurses, because provider champions had limited time to spend with patients. They suggested using PDSA cycles to identify strategies for role expansion or spreading the change in role to additional sites:

[PDSA cycles can be used] to expand staff roles and responsibilities, such as having the MAs [medical assistants] ask patients to remove shoes as a prompt for foot exam. — CHC 1, team leader

We have redesigned our system, training MAs to do finger sticks, point-of-service testing. We are considering this system redesign for our satellite site. — CHC 13, team leader

### Clinical information

All of the CHC team members we interviewed recognized the importance of using a patient registry to review clinical outcomes and improve care. Depending on the CHC's information technology (IT) resources, teams used different methods to track patient outcomes. Each CHC created a registry of diabetic patients in its population of focus either via an electronic medical record or the Patient Electronic Care System (PECS), a widely used chronic disease registry. Producing regular reports with patient data generated interest from providers and others within the CHC:

In the first 6 months of the collaborative, when we saw our data, all our numbers were low. We recognized that we were not lab testing our patients, especially regarding HbA1c [hemoglobin A1c]. Our provider champion began sending his patients for labs, including HbA1c, microalbumins. We had never even done microalbumins before. Our medical director would give a report at provider meetings, and when the other providers began to see results they became more aggressive in treating their diabetic patients. —  CHC 4, team leader

The patient registry also generated some negative feedback, for example, that it lacked IT support, that the PECS or electronic medical record was limited, and that extra resources were necessary to gather and enter data into the registry:

It is very important to have an IT person, a good IT system, and people who have knowledge about IT. We need to have more detailed discussions on mechanics of data collection prior to the project's startup. — CHC 14, team leader

### Organization

Promoting buy-in and support was a major theme for the organization component and had 3 dominant subthemes: senior leadership, provider champions, and staff. Making changes in the CHC was perceived to be difficult without the endorsement of CHC leadership:

Senior leadership buy-in, such as the CEO, is very important, with an emphasis on getting this buy-in early on in the project. The best thing that happened was at the kickoff for the collaborative, CEOs attended. If you want to make changes, you need senior leadership to make it a requirement. — CHC 7, team leader

At some CHCs, the provider champion advocated for the team, soliciting additional resources and assistance:

We continue to struggle. Our provider champion went to senior management and has been able to add an additional member to our team 2 months ago, a medical assistant. We hope to have less problems as staff has increased. — CHC 10, team leader

Without an engaged and active provider champion, reorganization was more difficult. Similarly, educating staff about the collaborative can prevent resistance from non-team members.

Another dominant subtheme for success in the organization component was changing the organization's policies and procedures. Some teams said their members worked with human resources staff to modify job descriptions and responsibilities. In addition, some sites altered how all CHC patients are seen:

We have now made it policy that our diabetic patients have an HbA1c every 3 months, cholesterol annually, and microalbumin. We have also begun educating our patients to test [blood glucose] 2 hours after their biggest meal rather than testing in the morning only. — CHC 3, team leader

### Community

CHC teams reported that linking with the community sometimes required pooling resources by working with other agencies, including other CHCs:

We were involved in another grant where we were meeting with other CHCs. This allowed us to identify other resources and use these connections. — CHC 11, team leader

### Additional themes

We grouped responses that did not fit into the Chronic Care Model components into a separate category. Additional themes for this category were staffing issues, time constraints, persistence, and administrative support from the League of Community Health Centers.

Staffing issues included staff turnover, loss, and a lack of staff to assume the additional responsibilities of the collaborative. An additional barrier was limited time available to meet regularly as a health care team or to generate reports required for the collaborative. These 2 major challenges, in addition to issues with the patient registry, were universally reported by teams as challenges to achieving success in the collaborative. Team members also said that persistence was necessary to achieve the collaborative's goals. Several team members expressed the need to "keep trying and not give up" despite the additional work generated by participating in the collaborative. Most teams reported that the assistance, support, and encouragement from the League of Community Health Centers facilitated success in the collaborative, particularly information provided at the learning sessions and the monthly feedback on reports by the league's quality initiatives manager.

### PDSA cycles

CHC teams described the type and number of PDSA cycles completed, by Chronic Care Model component each month. The most common activities addressed delivery system design. The least common were related to community (Figure).

**Figure.**
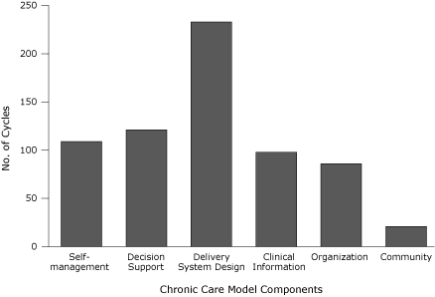
Number of plan-do-study-act (PDSA) cycles reported by community health center teams, Massachusetts Diabetes Health Disparities Collaborative, 2006.

**Table:** 

**Self-management**	**Decision Support**	**Delivery System Design**	**Clinical Information**	**Organization**	**Community**
109	121	233	98	86	21

## Discussion

This study confirms and expands findings from previous research (6,12,13) regarding the challenges encountered by CHCs. We identified several facilitators for achieving success in a health disparities collaborative: a paradigm shift to involve the patient in goal setting and decision making, learning sessions that deliver information about guidelines, IT support to develop and use patient registries, support from key leaders in the organizations, and linking with other CHCs to share resources. Teams noted that persistence was essential to achieve success across all areas of the model.

Teams concurred that CHCs may need to change their approach to health care delivery to increase the percentage of patients with documented self-management goals. Helping patients to become engaged in their own care, and have clear self-management goals, requires that the health care team understands the patient's priorities and concerns. Making self-management goal setting a team priority complements this change in approach because all members of the team must help patients to set realistic and achievable goals and provide encouragement and positive reinforcement when goals are met.

CHC staff consistently described their patient data registry as both a barrier to and a facilitator of success. Despite recognizing the benefits of having patient data to examine outcomes, track progress, and engage providers, almost all sites struggled with their patient registry. Chin et al (6) described similar findings. Teams said they needed training before starting and needed ongoing IT support during the course of the collaborative. For organizations considering convening a health disparities collaborative, recognizing this need and providing IT training and resources before implementation could improve progress toward collaborative goals. Ideally, staff at CHCs would then have more time to use data from the registry to tailor interventions for improvements rather than struggling during the first few months to populate their registries.

CHCs rarely made changes related to the community component of the Chronic Care Model (Figure). Two previous studies (10,20) explored the number and intensity of collaborative activities as measured by PDSA cycles. One study (20) reported findings similar to ours; the mean number of PDSA activities in the community component of the Chronic Care Model ranked lowest. In contrast, Grossman et al (10) reported a high number of PDSA activities related to community, but the activities were low intensity, as measured by the likelihood of improvement as an outcome of the intervention. Although it is unclear why connection with the community was the component with the least reported activity in our study, CHCs have a long history of community partnership and involvement. Thus, they may not attempt to form new partnerships because they are already engaged with their communities. Teams reported that time was a barrier to participation in the collaborative, and establishing new community relationships can be time-consuming. Moreover, engaging CHCs in community partnerships may be beyond the scope of work for team members and may need to be addressed by senior management. Future collaboratives may wish to encourage participating CHCs to re-establish community partnerships or conduct outreach to new organizations.

These findings must be considered in light of the limitations of the study. First, the interviews were conducted with CHCs in a single state, so the findings may not be generalizable to all states. However, the CHCs that participated in the collaborative represented diverse patient populations from both urban and rural locations. Second, CHCs that participated in the collaborative chose to do so and, therefore, may differ from nonparticipants in terms of resources, staff, and patients.

We used a rigorous qualitative method to describe and categorize the experiences of CHCs that participated in a health disparities collaborative. Our taxonomy, based on the Chronic Care Model, proved successful, as determined by intercoder agreement, and may be useful in qualitative measurement of other quality improvement efforts.

## Figures and Tables

**Table 1:** Characteristics of Patients at Community Health Centers Compared With Patients Statewide, Massachusetts Diabetes Health Disparities Collaborative, 2006

**Characteristic**	Community Health Centers, %^a^,^b^	Massachusetts, %^b^
**Race/ethnicity^c^**		
Non-Hispanic white	32	79
Non-Hispanic black	23	7
Hispanic	33	9
Other	5	5
Missing	8	0
**Health insurance status^d^**		
Private	20	66
Medicare	7	15
Medicaid	36	17
Other	2	0
None	35	3

a Reflects data from 15 of the 17 participating community health centers.

b Percentages may not total 100 because of rounding.

c Reference 18.

d Reference 19.

**Table 2:** Themes of Activities, Massachusetts Diabetes Health Disparities Collaborative, 2006

**Overarching Theme^a^**	Subtheme
**Self-management** Provide basic information to patients about their disease. Assist patients with self-management skill building. Provide ongoing support.	• Changing practice to make self-management a priority • Actively involving patients in self-management
**Decision support** Integrate explicit, proven guidelines into practice. Provide ongoing education to providers.	• Understanding and implementing guidelines
**Delivery system design** Clarify roles and tasks to ensure patients get needed care. Plan visits based on the patient's needs.	• Language and literacy • Roles and responsibilities: changing or assigning tasks to team members
**Clinical information ** Use the registry to guide treatment, anticipate problems, and track progress.	• Benefits of the registry: reviewing clinical outcomes and improving care • Challenges of the registry: time, access to data, and data entry
**Organization** Integrate efforts to improve care into the organization. Engage entire organization in the improvement effort. Ensure that senior leaders and provider champions are visible and committed members of the team.	Promoting buy-in and support: • Senior leadership: support from leadership • Provider champions: support from provider champions • Staff: training or educating staff at the center Changing the organization's policies and procedures: changing the organizational structure by working with human resources to change job descriptions or altering the way patients are seen
**Community** Form associations and partnerships with state and local agencies, educational institutions, religious organizations, businesses, and clubs.	• Linking with agencies: connecting with other agencies • Linking with projects: using other projects or grants to extend services
**Additional themes**	• Staff: loss of staff, lack of adequate staff, or limited staff • Time: time constraints and lack of time to meet • Persistence: continuing to work on the collaborative • Administration: assistance, support, or encouragement from the administrating agency

a Definitions of components of the Chronic Care Model taken from Institute for Healthcare Improvement (5).

## Appendix. Interview Questions for Community Health Center Teams, Massachusetts Diabetes Health Disparities Collaborative, 2006

1. What do you think has been the most significant change in patient care that has occurred at your community health center because of participation in the collaborative? How? What? Why?
2. What strategies did you employ to achieve this change?
3. What challenges or obstacles have you encountered? Probe: Have there been challenges related to leadership, provider involvement, staffing, other resources, time?
4. How did you handle the challenges you identified?
5. Are there any take-home points that you would share with other community health centers that may be encountering challenges?
6. Do you have any additional comments you would like to share regarding your experiences in the Diabetes Health Disparities Collaborative?
